# e-Health Ethics Draft Code (Feb 18)

**DOI:** 10.2196/jmir.2.1.e2

**Published:** 2000-02-29

**Authors:** Helga Rippen, Ahmad Risk

**Keywords:** Internet, Ethics, Quality of Health Care

## Abstract

The Internet is changing how people receive health information and health care. All who use the Internet for health-related purposes must join together to create an environment of trusted relationships to assure high quality information and services; protect privacy; and enhance the value of the Internet for both consumers and providers of health information, products, and services. The goal of the "e-Health Code of Ethics" is to ensure that all people worldwide can confidently, and without risk, realize the full benefits of the Internet to improve their health. The draft code, presented in this paper, has been prepared as a result of the "eHealth Ethics Summit," which convened in Washington DC on 31 January 2000 - 2 February 2000, organized by the Internet Healthcare Coalition and hosted by the World Health Organisation/Pan-American Health Organisation (WHO/PAHO), and attended by a panel of about 50 invited experts from all over the world. It sets forth guiding principles under five main headings: candor and trustworthiness; quality; informed consent, privacy, and confidentiality; best commercial practices; and best practices for provision of health care on the Internet by health care professionals.

*Please note: This is a discussion paper. Feedback on this proposal may be given until April 14, 2000. (Please use the feedback form at http://mednetics.org/ethics/ehes_feedback.htm)*

*Note added on May 24, 2000: This draft is now obsolete. The **final version** of this article has been published in the Journal of Medical Internet Research 2000;2(2):e9.*

## Vision Statement

The Internet is changing how people receive health information and health care. All who use the Internet for health-related purposes must join together to create an environment of trusted relationships to assure high quality information and services, protect privacy, and enhance the value of the Internet for both consumers and providers of health information, products, and services. The goal of the "e-Health Code of Ethics" is to ensure that all people worldwide can confidently, and without risk, realize the full benefits of the Internet to improve their health.

## Introduction

Health information has the potential both to improve health and to do harm. All people who use the Internet for health-related purposes must be able to trust that the sites they visit adhere to the highest ethical standards and that the information provided is credible.

Because health and health care are critically important to people, the organizations and individuals that provide health information on the Internet have special, strong obligations to be trustworthy, provide high quality content, protect users' privacy, and adhere to standards of best practices for online commerce and online professional services in health care.

## Guiding Principles

**1. Candor & Trustworthiness**

Guiding Principle:

Organizations and individuals providing health information, products, or services on the Internet have an obligation to candidly disclose

(A) those factors that could influence content

(B) the potential risks of providing personal information on the Internet

**2. Quality**

Guiding Principle:

Organizations and individuals offering health information, products, or services on the Internet have an obligation to

(A) provide high quality information, products, or services

(B) provide means for users to evaluate the quality of health information

**3. Informed Consent, Privacy & Confidentiality**

Guiding Principle:

Organizations and individuals providing health information, products, or services on the Internet have an obligation to

(A) safeguard users' privacy

(B) obtain users' informed consent when gathering personal information

**4. Best Commercial Practices**

Guiding Principle:

Organizations and individuals who sponsor, promote, or sell health information, products, or services on the Internet have an obligation to

(A) disclose any information a reasonable person would believe might influence his or her decision to purchase or use products or services

(B) be truthful and not deceptive

(C) engage in responsible business relationships and affiliations

(D) guarantee editorial independence

(E) disclose the site's privacy policy and terms of use

**5. Best Practices for Provision of Health Care on the Internet by Health Care Professionals**

Guiding Principle:

Health care professionals and organizations who provide health information, products, or services on the Internet have an obligation to

(A) adhere to the highest standards of professional practice

(B) help patients to understand how the Internet affects the relationship between professional and patient while adapting the highest professional standards to the evolving interactions made possible by the Internet

## Definitions

Health Information

Health information includes information for staying well, preventing and managing disease, and making other decisions related to health and health care.

It includes information for making decisions about health products and health services.

It may be in the form of data, text, audio, and/or video.

It may be subject to frequent changes due to the updating of websites or user-provider interactivity

**1. Candor & Trustworthiness**

Guiding Principle: Organizations and individuals providing health information, products, or services on the Internet have an obligation to candidly disclose

(A) those factors that could influence content

(B) the potential risks of users providing personal information on the Internet

Principle Part (A): Content

People who use the Internet for health-related purposes must have sufficient information to make fully informed decisions about the integrity of content and the potential for bias. Thus

Organizations and individuals offering health information, products, or services on the Internet have an obligation to

(1) prominently, clearly, and accurately indicate

(a) ownership of the site or service

(b) the purpose of the site or service

(c) how to contact the owner and the party responsible for the site or service

(d) any relationship, financial or other, that a reasonable person would believe might influence the user's perception of the information, products, or services offered

(2) clearly distinguish advertising from educational or scientific content

Principle Part (B): Risk

People may not realize that personal information may be collected when they use the Internet. And they may not understand that declining to provide personal data may affect the information or services they receive. Thus

Organizations and individuals offering health information, products, or services on the Internet have an obligation to

(1) alert users to the potential risks to the privacy of personal information on the Internet (for example, that third parties may be collecting information without the site's knowledge)

(2) provide clear, complete, and accurate information regarding

(a) what information is being collected and by whom

(b) to what uses information will be put

(c) the possibility that information will be distributed to/acquired by third parties

(d) the choices available to the individual regarding use and distribution

(e) steps to data quality and access

(3) state the organization's commitment to data security

(4) clearly disclose the consequences, if any, of refusing to provide personal information

(5) clearly describe the accountability mechanism used by the organization or site and how to contact the responsible party

**2. Quality**

Guiding Principle: Organizations and individuals offering health information, products, or services on the Internet have an obligation to

(A) provide high quality information, products, or services

(B) provide means for users to evaluate the quality of health information

Principle Part (A): Quality

People who use the Internet for health-related purposes need credible, well-supported information in order to make prudent decisions. Thus

Organizations and individuals offering health information, products, or services on the Internet have an obligation to provide information that is

(1) culturally appropriate and easy to use

(2) accurate and unbiased

(3) up to date

Further explanation of Part (A)

(1) High quality health information

(a) uses language that is culturally appropriate for intended users

(b) presents information in language that is easy to read

(c) presents information in formats that accommodate the needs of special populations (for example, large type for users who are visually impaired)

(2) High quality health information should

(a) be rigorously and fairly evaluated

(b) be consistent with the best available evidence

(c) distinguish "experience-based" information from information that has been formally evaluated scientifically

(d) present all reasonable sides of controversial issues

(3) High quality health information should clearly display

(a) publication date and version number (if appropriate)

(b) date last reviewed

(c) date when substantive changes were last made

Principle Part (B): User Evaluation

People who use the Internet for health-related purposes need to be able to judge the credibility of content. Thus

Organizations and individuals offering health information, products, or services on the Internet have an obligation to

(1) clearly and accurately

(a) disclose the sources of information

(b) disclose how the site evaluates information

(c) indicate when there have been substantive changes in the information

(2) provide tools for feedback from users about the quality of content and usability of the site

**3. Informed Consent, Privacy & Confidentiality**

Guiding Principle: Organizations and individuals providing health information, products, or services on the Internet have an obligation to

(A) safeguard users' privacy

(B) obtain users' informed consent when gathering personal information

Principle Part (A): Informed Consent

To make prudent decisions about whether to provide personal information online, especially information about their health status, people need to know what information is being gathered and why. Thus

Organizations and individuals providing health information, products, or services on the Internet have an obligation to

(1) prominently and clearly describe

(a) what information is being gathered

(b) how that information will be used (for example, for individual health care, research, or commercial purposes)

(c) with whom information will be shared

(d) how/where information will be stored and for how long it will be stored

(2) verify that users have given their voluntary informed consent to collect and use personal information in the ways described

Principle Part (B): Privacy and Confidentiality

The personal information that may be gathered by a health-related site is often intimate and highly sensitive. People must be able to trust that any personal information they provide will be kept confidential and secure. Thus

Organizations and individuals providing health information, products, or services on the Internet have an obligation to

(1) prevent unauthorized access to personal information

(2) assure users' access to their personal information

(3) assure users' rights to review personal information and to amend it as appropriate or necessary

(4) provide mechanisms for tracing use of personal information (for example, audit trails)

**4. Best Commercial Practices**

Guiding Principle: Organizations and individuals who sponsor, promote, or sell health information, products, or services on the Internet have an obligation to

(A) disclose any information a reasonable person would believe might influence his or her decision to purchase or use products or services

(B) be truthful and not deceptive

(C) engage in responsible business relationships and affiliations

(D) guarantee editorial independence

(E) disclose the site's privacy policy and terms of use

Principle Part (A): Transparency

People who use the Internet for health-related purposes need to be assured that commercial health or medical sites are trustworthy. They have a right to expect that material presented as scientific or educational in nature is accurate, timely, and objective, and to be assured that they will be able to choose, consent, and control when and how they actively engage in a commercial relationship. Thus

Organizations and individuals who sponsor, promote, or sell health information, products, or services on the Internet have an obligation to

(1) prominently, clearly, and accurately identify the business and/or site sponsors

(2) clearly distinguish content intended to promote or sell a product, service, or organization from educational or scientific content

(3) clearly disclose any financial or other incentives for providers who develop or present content

Principle Part (B): Truthfulness

People using the Internet for health-related purposes need to know that products or services are described truthfully and that information is accurate and reliable. Thus

Organizations and individuals who sponsor, promote, or sell health information, products, or services on the Internet have an obligation to

(1) Tell the truth; tell the whole truth; make sure it is the truth. Thus they must [gk]

(a) be truthful and not deceptive in all content used to promote the sale of health products or services

(b) clearly disclose information that if known by consumers would likely affect the consumer's purchase or use of a product or service

(c) be truthful in any claims about the efficacy, performance, or benefits of products or services

Principle Part (C): Business Relationships & Affiliations

People who use the Internet for health-related purposes must be confident that commercial sites select partners who adhere to the highest ethical standards. Thus

Organizations and individuals who sponsor, promote, or sell health information, products, or services on the Internet have an obligation to

(1) make reasonable efforts to ensure that linked and partner sites comply with applicable law and uphold the same ethical standards as the site itself

(2) encourage users who believe that a site's commercial partners or affiliates may violate law or ethical principles to notify the site's webmaster

Principle Part (D): Editorial Independence

People who use the Internet for health-related purposes must be able to clearly distinguish editorial content from content intended to promote or sell health products or services. Thus

Health-related sites supported by advertising or other commercial sponsorship have an obligation to

(1) clearly separate advertising from editorial process and

(2) assure that the site's editors have full authority for determining editorial content

(3) assure that the site's editors have sole discretion to determine the type of advertising that will be accepted and full authority to reject advertising of any kind

(4) assure that current or potential sponsors do not influence the way search results for specific information on key words or topics are displayed.

Principle Part (E): Privacy

See 3. Privacy, above.

**5. Best Practices for Provision of Health Care on the Internet by Health Care Professionals**

Guiding Principle: Health care professionals and organizations who provide health information, products, or services on the Internet have an obligation to

(A) adhere to the highest standards of professional practice

(B) help patients to understand how the Internet affects the relationship between professional and patient while adapting the highest professional standards to the evolving interactions made possible by the Internet

Principle Part (A): Professional Standards

Health care professionals have fundamental ethical obligations to patients. Thus

Physicians, nurses, pharmacists, therapists, and all other health care professionals who provide information, products, or services pertaining to an individual's health care on the Internet have an obligation to

(1) serve patients' best interests

(2) protect patients' confidentiality (by adhering to the principle of privacy discussed above)

(3) conscientiously assess patients' needs and local resources in order to recommend or provide appropriate health information or services

(4) abide by the ethical codes governing their professions as practitioners in face-to-face relationships

(5) obey the laws and regulations of the relevant jurisdiction

Principle Part (B): The Professional-Patient Relationship

The Internet can be a powerful tool for helping to meet patients' health care needs, but it also has limitations. Thus

Health care professionals who practice on the Internet have an obligation to

(1) clearly and accurately describe the nature of the online relationship

(a) identify themselves, their location, and their professional credentials

(b) stress that it is important for patients to identify themselves and describe their health care needs as clearly they can

(2) clearly and accurately describe the constraints of online diagnosis and treatment recommendations (for example, that the professional cannot prescribe certain medications online)

(3) describe the nature of information being provided (for example, whether based on expert consensus, personal professional judgment, or other sources of evidence)

(4) help "e-patients" understand that although not every aspect of health care requires a face-to-face interaction, online consultation should not take the place of an ongoing relationship with a health care provider in every situation [bc]

(5) clearly disclose any sponsorships, financial incentives, or other information that might affect the professional's role or the services offered

(6) clearly disclose how payment for services is to be made

